# Adaptive Circadian Rhythms for Autonomous and Biologically Inspired Robot Behavior

**DOI:** 10.3390/biomimetics8050413

**Published:** 2023-09-06

**Authors:** Marcos Maroto-Gómez, María Malfaz, Álvaro Castro-González, Sara Carrasco-Martínez, Miguel Ángel Salichs

**Affiliations:** Systems Engineering and Automation, University Carlos III of Madrid, Av. de la Universidad 30, 28911 Leganés, Madrid, Spain; mmalfaz@ing.uc3m.es (M.M.); acgonzal@ing.uc3m.es (Á.C.-G.); sacarras@ing.uc3m.es (S.C.-M.); salichs@ing.uc3m.es (M.Á.S.)

**Keywords:** biological rhythms, robotics, artificial intelligence, autonomous and adaptive behavior, social robotics

## Abstract

Biological rhythms are periodic internal variations of living organisms that act as adaptive responses to environmental changes. The human pacemaker is the suprachiasmatic nucleus, a brain region involved in biological functions like homeostasis or emotion. Biological rhythms are ultradian (<24 h), circadian (∼24 h), or infradian (>24 h) depending on their period. Circadian rhythms are the most studied since they regulate daily sleep, emotion, and activity. Ambient and internal stimuli, such as light or activity, influence the timing and the period of biological rhythms, making our bodies adapt to dynamic situations. Nowadays, robots experience unceasing development, assisting us in many tasks. Due to the dynamic conditions of social environments and human-robot interaction, robots exhibiting adaptive behavior have more possibilities to engage users by emulating human social skills. This paper presents a biologically inspired model based on circadian biorhythms for autonomous and adaptive robot behavior. The model uses the *Dynamic Circadian Integrated Response Characteristic* method to mimic human biology and control artificial biologically inspired functions influencing the robot’s decision-making. The robot’s clock adapts to light, ambient noise, and user activity, synchronizing the robot’s behavior to the ambient conditions. The results show the adaptive response of the model to time shifts and seasonal changes of different ambient stimuli while regulating simulated hormones that are key in sleep/activity timing, stress, and autonomic basal heartbeat control during the day.

## 1. Introduction

**Figure 1:**
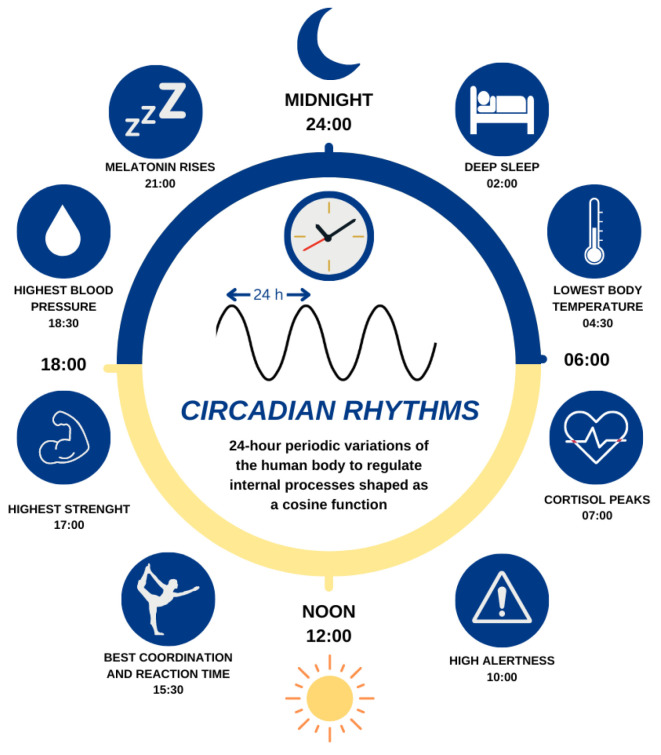
The circadian clock and its primary related functions.

Since ages, those living beings with better adaptive capabilities have demonstrated more chances to survive and genetically evolve in new generations [1]. In the XVIII century, Jean Jacques d’Ortous de Mairan [2] discovered that one of the leading causes of living beings’ adaptation was biological rhythms, periodic variations in their internal state adapted to ambient conditions [3]. Recent findings [4] highlight that the suprachiasmatic nucleus (SCN) is the primary pacemaker in humans. The SCN is a brain region synchronized and entrained with photic information associated with the Earth’s rotation (24 h) that controls many biological functions, as Figure 1 shows. Other studies [5,6] show that secondary rhythms are also present in peripheral tissues along the human body (like the adrenal glands), implicated in important functions controlled by the SCN.

Artificial intelligence researchers have shown interest in providing robots with biologically inspired behavior to mimic human functions like emotion, motivation, or cognition. These studies aim to develop robots with human-like skills to interact with people [7] successfully. This research line hatched in the early 2000s, endowing robots with adaptive and affective behavior to improve the robots’ expressiveness, liveliness, and naturalness [8]. However, nowadays, most robots carry out predefined tasks under controlled conditions, not including adaptive mechanisms to overcome unexpected environmental changes. For this reason, the robotic community challenges the design of systems that autonomously recognize environmental changes and adapt to perform better. Besides, endowing robots with human-like social skills facilitates Human-Robot Interaction (HRI) since the robots’ communicative mechanisms are akin to those exhibited by humans [9].

This paper presents a biologically inspired adaptive model for autonomous robots based on replicating humans’ biological rhythms to operate for long periods overcoming ambient changes. The proposed mechanism mimics the circadian clock in the human SCN to adapt the robots’ behavior to ambient conditions. Recently, An et al. [10] presented *dCiRC* (Dynamic Circadian Integrated Response Characteristic) as a dynamic method for generating reactive circadian signals to light profile. dCiRC updates the initial proposal *CiRC* by Roenneberg et al. [11], integrating the dynamic response that living beings genetically possess to light. Taking advantage of this methodology, our model includes the adaptation of dCiRC to other ambient stimuli like ambient noise or the robot’s users’ activity as a new contribution. Including this adaptive mechanism provides more personalized support to users when they are more active and require assistance. In this manuscript, we show the application of the dCiRC model and the modeling of human biological functions to generate autonomous and adaptive robot behavior for long periods.

The dCiRC circadian signal emulates a biological clock that regulates the periodicity of the artificial biological functions emulated in the robot. Thus, these functions react to the external environment, adapting the robot’s behavior as humans do. The method uses the robot sensors to capture ambient information and provide a reactive signal that adapts the robot’s behavior. The goal of the proposed method is to use an adaptive circadian clock to synchronize the robots’ functions, such as its activity periods (e.g., sleep when its user sleeps) or biologically inspired processes (e.g., synchronize the robot’s expressiveness like its heartbeat) to the environmental condition and the activity periods of their users. This application aims to provide more adapted and accurate assistance, making users perceive the robots as more natural, competent, and intelligent, as previous evaluations with robot users reveal [12,13].

The results presented in this paper show the system’s performance and possibilities of adapting to different ambient conditions and social environments. The objectives of applying our model to robots are various. First, we show the capacity of the dCiRC model to shift the clock’s timing to match the light h so the robot does not overwhelm the user with continuous activity and assistance by going to sleep during the night or in the h the user is sleeping. Furthermore, we use the clock’s ability to expand and compress its cycle length to adapt to seasonal changes to expand the robot’s activity to match that of their users by considering the users’ activity as the primary external stimulus with influence on our circadian rhythm (called *zeitgeber marker* in the literature). The regulation of its internal rhythms allows the robot to expand (e.g., during the summer when light h expand) or compress (e.g., during winter) its activity h so assistance is provided in appropriate metrics. Besides, we provide examples of how our biological model autonomously controls the robot’s voluntary behavior by adapting some functions to execute appropriate behaviors reacting to changing ambient conditions. These examples show the robot’s sleeping behavior adapted to ambient conditions and how involuntary functions such as stress or the heartbeat evolve by reacting to ambient stimuli to dynamically adapt the robot’s expressiveness and behavior to facilitate assistance and HRI.

This paper is organized as follows. Section 2 analyzes similar works that previously explored integrating biological rhythms in robots. Section 3 presents dCiRC and our model based on biological rhythms to attain autonomous adaptive behavior in robotic systems. Section 4 describes the scenario and parameters to run the model. Section 5 shows how the biological clock controls the robot’s biological processes, shifting their natural rhythms to exhibit adaptive behavior and maintain the robot’s condition in a good state. Section 6 discusses the results of this work, and Section 7 provides new insights into the importance of adaptive behavior in robotic systems using biologically inspired concepts. Finally, Section 8 contains the main findings of this work.

## 2. From Human to Robot Biological Rhythms

Autonomous adaptive behavior is an intrinsic feature of living beings. The most intelligent animals, humans, can even act not only considering their inherited physiological needs but reasoning about the consequence of their actions [14]. In this context, endowing robots with biologically inspired behavior is framed as a modeling method to replicate human behavior on artificial systems to improve their naturalness and HRI capabilities. The following sections delve into how biological rhythms emerge in humans and review the literature applying this biology to robots.

### 2.1. Human Biological Rhythms

Circadian rhythms are periodic 24-h variations that control critical biological functions in living beings such as emotion, body temperature, or alertness [15]. These rhythms are not only a consequence of external conditions since many studies have reported that organisms possess endogenous self-sustained rhythms even in the absence of arousing stimuli [3,15,16]. The previous studies reveal the presence of an endogenous circadian rhythm that controls our bodies. The primary circadian pacemaker in humans is the SCN, a brain region connected to the eyes via the retino-hypothalamic tract [17]. Due to this connection, light is considered the primary influence on circadian rhythms, and many studies show its influence on shifting them [3,17]. Additionally to light, other external cues like social interaction, ambient noise, or physical activity affect circadian rhythms, defining our decision-making and adaptive behavior [18].

As the systematic review carried out by Asgari-Targhi & Klerman [19] reports, in the last decades, many different approaches have been considered to mathematically shape the evolution of human circadian rhythms and the influence of external stimuli on them. The two most important streams in this scope are Phase Response Curves (PRC) and Velocity Response Curves (VRC), which consider the light profile the most important external cue that causes time shifts in the circadian clock.

In the last decade, the proposal by Roenneberg et al. [11] gained massive attention as a PRC model since it does not make assumptions about the dynamics of time shifting, proving good adaptation under all conditions. This model was updated by An et al. [10] to combine the benefits of PRC with the velocity changes of VRC. As we analyze later, we opted to use An et al.’s model in our biologically inspired system to generate dynamic circadian rhythms and time shifts to control the artificial biology of robots.

### 2.2. Biological Rhythms in Robots

Several works have attempted to replicate biological rhythms in robots in the last few years. Although some works have been applied to robotics to control specific functions periodically [20], a few of them focus on using biological rhythms to control long-term autonomous robot behavior. Next, we review the works up to date in this research line.

The first study using circadian oscillators to control autonomous robots dates from 2001. In this work, Arkin et al. [21] presented an ethological and emotional model for the robot AIBO. The model controls the biologically inspired functions to drive the robot’s behavior, which is also affected by a dimensional emotional space. Although circadian rhythms are mentioned to affect the arousal dimension of the emotional space, it is not defined its real influence. Later, in 2004, Stoytchev & Arkin [22] continued in this research line and developed a motivational model for the autonomous behavior of robots. In this work, circadian rhythms drive motivation, such as sleep or hunger, to be elicited in a cyclic manner peaking at certain h. The study presents circadian rhythms as periodic signals that affect motivation. However, the authors do not present their mathematical foundation.

Sauzé & Neal [23] designed in 2008 an artificial neuroendocrine system for the autonomous behavior and survival of sailing robots. Their model emulates hormones to control the robot’s behavior. Regarding circadian rhythms, a day/night hormone presents higher values during the day, enabling more behaviors used to control the robot’s activity. The rhythm exhibited by this hormone is represented as a cosine signal, being the first work found in the literature with a certain degree of similarity with human rhythms. However, this work uses a fictional hormone, and the adaptation of circadian rhythms is not considered. Like the previous authors, Burratini & Rossi [24] developed in 2008 a robotic architecture to control the autonomous behavior of robotic systems. Circadian rhythms play an important role in activating behavior and dynamically modify the accessibility to the robot’s sensors depending on the environment’s condition. Again, the major shortcoming of the work is not considering the dynamic nature of circadian time and the influence of these rhythms on other biological processes of the robot.

In 2018, O’Brien & Arkin [25] developed a circadian system for robots to adapt their behavior selection depending on a model that the system does from the environment. Behaviors are activated depending on a weighted function that considers the circadian rhythm, the robot motivation, and releasing mechanisms shaped by external perceptions. The model represents essential factors of circadian rhythms, like their cycle length and the influence of external factors but lacks in defining their shifting. Besides, circadian rhythms directly affect behavior selection, missing the important role of other biological functions like neuroendocrine substances or biological processes.

More recently, Ito et al. [26] presented in 2022 a biologically inspired model for implementing intelligent functions in soft robotics. Their work considers circadian rhythms as 24-h periodic variations that influence biological functions. Although biological rhythms are addressed from a purely biological perspective, the paper only presents the initial insights of a research work that does not explain the relationship between the rhythms and the robot’s behavior.

Recently, we [27] explored the role of modeling involuntary behavior on social robots. In this work, autonomic processes such as heart rate or pupil size vary depending on the robot’s perceived stimuli and their influence on artificial neuroendocrine substances. The biological processes integrated into the model have circadian rhythms that evolve along the day, making the robot change its expressions depending on the time of the day and the stimuli. As in the previous examples, the model considers external stimuli to change the intensity of the robot’s functions but does not consider the power of stimuli to shift circadian timing and change the robot’s behavior.

The literature review reveals that circadian rhythms have been used in robotics to elicit periodic functions that drive behavior. Besides, some of these works state that biologically inspired behavior improves the robot’s naturalness and other robot attributes during HRI. However, these contributions do not consider the importance of external stimuli to change the biological rhythms of the robot to adapt and anticipate environmental changes, making the robot’s behavior predictable and repetitive. For better HRI, responsiveness, naturalness, and behavior selection of robots operating in changing ambient conditions, using accurate biological models like dCiRC is essential. For this reason, our contribution to the area is to propose a method for generating biologically inspired adaptive behavior in robots using the dCiRC model to produce the dynamic biorhythms that drive decision-making.

(1) $$CiRC\left(\theta_{I}\right)=\left\{\begin{array}{cc} & \mathrm{when}\;0<\theta_{I}<\pi ,\\ \sin\left(\theta_{I}\right)+s\times \sin\left(2\theta_{I}\right) & \mathrm{if}\;CiRC<0\;,\;\mathrm{then}\;CiRC=0\\ & \mathrm{if}\;a>1\;,\;\mathrm{then}\;CiRC=CiRC\times a\\ & \mathrm{when}\;\pi <\theta_{I}<2\pi \\ -\sin\left(2\pi -\theta_{I}\right)-s\times \sin\left(2\pi -2\theta_{I}\right) & \mathrm{if}\;CiRC>0\;,\;\mathrm{then}\;CiRC=0\\ & \mathrm{if}\;a>1\;,\;\mathrm{then}\;CiRC=CiRC/a \end{array}\right.$$

(2) $$ODEs\left\{\begin{array}{c} \frac{d\theta_{Z}}{dt}=\frac{2\pi }{T}\\ \frac{d\theta_{I}}{dt}=\omega_{I}\\ \frac{d\omega_{I}}{dt}=cz\times \mathrm{External}\;\mathrm{signal}\left(\theta_{Z}\right)\times \mathrm{CiRC}\left(\theta_{I}\right)+k_{e}\left(\omega_{I0}-\omega_{I}\right) \end{array}\right.$$

(3) $$\mathrm{External}\;\;\;\mathrm{signal}\left(\theta_{Z}\right)=\alpha \times \mathrm{light}\left(\theta_{Z}\right)+\beta \times \mathrm{noise}\left(\theta_{Z}\right)+\gamma \times \mathrm{activity}\left(\theta_{Z}\right)$$

## 3. Modeling Biological Rhythms

This section sets up the dCiRC model and how we have modified it to consider the light profile, ambient noise, and user activity as stimuli that influence the internal clock. Table 1 contains the abbreviations used by the model. The section presents the biological model where dCiRC regulates the robot’s daily rhythms. The model presented in this contribution addresses three application areas unexplored in robot behavior generation. First, it shapes the peaks and nadirs some neuroendocrine substances experience at specific moments of the day and how they affect voluntary behavior through motivation. Second, it investigates how circadian rhythms are generated to control involuntary behavior and autonomic processes like the heartbeat. Finally, it considers these rhythms’ time shifts and seasonal changes depending on internal and ambient stimuli emulating the humans’ biological clocks.

### 3.1. Dynamic Circadian Integrated Response Characteristic

The *Dynamic Circadian Integrated Response Characteristic* (dCiRC) model [10] is a unified method that represents the circadian rhythms that many living beings exhibit as a consequence of the light-dark periods. Unlike previous approaches in characterizing circadian rhythms [11], dCiRC considers gradual changes in the clock parameters due to ambient conditions (parametric models) and time shifts of the clock responding to the stimuli (non-parametric models). To represent the internal and external markers of the agent, the notation *I* and *Z* is chosen.

The method consists of adjusting the cycle length ($\tau_{I}$ in h) of the agent’s internal clock to match that of the external environment (*T* in h) at each specific time of the day *t* (h from the beginning of the trial expressed using a float number). The time of the day is expressed in radians as a phase angle $\theta_{Z}=t\times 2\pi /24$ normalized from 0 to 2π. Changes in the internal cycle length occur by dynamically adjusting the velocity of the clock ($\omega_{I}$ in radians per h). Depending on the ambient conditions, the cycle length ($\tau_{I}$) is expanded (decelerate $\omega_{I}$) or compressed (accelerate $\omega_{I}$), generating a circadian signal that acknowledges external changes and adapts to them. The change depends on the internal phase angle of the agent, expressed as $\theta_{I}$, that ranges [0,2π].

As Equation (1) shows, the circadian value ($CiRC(\theta_{I})$) consists of a sine curve and its first harmonic, evolving from −1 to 1. The shape factor (*s*) determines the influence of the harmonic on the circadian value and represents the extent of the dead zone around noon. Besides, an asymmetry factor (*a*) defines the ratio between the amplitudes of the compression and expansion areas. By definition, in the original model, the shape and asymmetry factors range from 0 to 2 units.

The dynamic component of the dCiRC model proposed in [10] employs three ordinary differential equations (ODEs), represented in Equation (2), that consider changes in the phase of the ambient condition ($\theta_{Z}$) to shift the phase of the clock ($\theta_{I}$) by regulating the clock’s velocity ($\omega_{I}$). Velocity changes in each time step depending on the intensity of external cues (External signal ($\theta_{Z}$)) and the CiRC value ($CiRC(\theta_{I})$) modulated by a factor cz representing the stimuli strength and impact. Furthermore, an elastic factor ($k_{e}$) corrects the differences between the actual clock velocity and the angular velocity of the internal clock in constant darkness ($\omega_{I0}$).

The initial version of the model considers the light profile as the stimulus used to adapt the cycle length. However, other ambient conditions such as noise, social relationships, or activity shift and modify the internal clock [3]. This paper proposes as one of its main contributions the Equation (3) to shape the External signal (External signal ($\theta_{Z}$)), that considers the light profile, ambient noise intensity, and activity to adapt the robot’s circadian rhythm to the ambient stimuli. The light, ambient noise, and activity profile range from 0 to 1 units and are obtained from the robot’s perceptions. The parameters α, β, and γ respectively weigh the influence of each stimulus on the external cues to balance how the robot considers ambient conditions. These parameters range from 0 to 1 and must sum 1 unit. It is worth mentioning that the External signal (Equation (3)), the three ODEs (Equation (2)), and the CiRC value are updated every time step *t* set to 0.5 seconds to produce the dynamic response of the circadian clock.

### 3.2. Biologically Inspired Model

**Figure 2:**
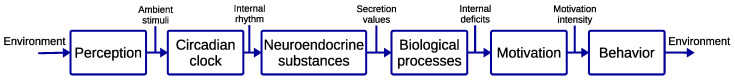
High-level block architecture proposed in this contribution.

The method presented in the previous section yields an adaptive circadian rhythm to ambient conditions able to compress and expand the cycle length ($\tau_{I}$) of the clock by dynamically changing its angular velocity ($\omega_{I}$), being $\omega_{I}=2\pi /\tau_{I}$. The adaptive circadian rhythm proposed in dCiRC enables new biologically inspired control strategies to produce autonomous robot behavior. This paper uses the previous method to contribute to an adaptive biological model, whose architecture is presented in Figure 2, for the autonomous behavior of robots operating and perceiving dynamic environments.

The biologically inspired model proposed in this paper considers a circadian signal generated by the dCiRC method to mimic human processes in the robot. Recent studies [28,29] suggest that the SCN is the primary pacemaker of the human body, exhibiting a circadian rhythm with a cycle length of around 24 h. The SCN projects to many regions in the brain and other body parts, influencing the regulation of physiological functions like sleep or stress and psychological processes like emotion and mood. Consequently, circadian rhythms exist at many levels of the human body, affecting neuroendocrine substances secretion and higher-level functions.

The model considers a circadian signal that emulates the role of the SCN in humans. The circadian rhythm regulates the secretion rates of specific neuroendocrine substances chosen to control robot functions and decision-making. Neuroendocrine substances are secreted in different body regions, showing circadian rhythms similar to a sine function, as the dCiRC proposes [30]. The rhythms that the secretion rates of these substances experience are typically time-shifted to the rhythm of the central pacemaker, as they are not secreted in the SCN. Considering this idea, we propose in this work that the strength of the time shift of the neuroendocrine substances, expressed as ($\theta_{n}$), depends on the distance between the SCN and where the substance is secreted. We propose Equation (4), drawing on [30], to represent how our biologically inspired model shapes the secretion rate of neuroendocrine substances ($ns(\theta_{Z})$),

(4) $$ns_{n}\left(\theta_{Z}\right)=\kappa_{n}\times CiRC\left(\theta_{I}+\theta_{n}\right)+0.01$$

where $\kappa_{n}$ determines the impact of the circadian rhythm on the neuroendocrine level and $\theta_{n}$ is the time shift value specific to each neuroendocrine substance.

Many studies have reported the essential role of neuroendocrine substances in regulating high-level biological processes. For example, melatonin induces sleep in the late evening [31], cortisol regulates stress levels [32], and oxytocin regulates social behavior [33]. These are just a few examples of the many relationships between neuroendocrine substances and high-level biological processes. We propose Equation (5) to mathematically define the value of a biological process ($bp_{p}$) in time $\theta_{Z}$,

(5) $$bp_{p}\left(\theta_{Z}\right)=bv_{p}+\lambda_{p}\times bp_{i}\left(\theta_{Z-1}\right)+\delta_{p}\times ns\left(\theta_{Z}\right)$$

where $bv_{p}$ is the reference basal value acting as the baseline of the process, $\lambda_{p}$ indicates if the process value accumulates between time steps and its value can be 0 or 1, $bp_{p}\left(\theta_{Z-1}\right)$ is the previous value of the process, and $\delta_{p}$ weighs the influence of the neuroendocrine substances on the process.

Biological processes evolve, showing circadian rhythms controlled by the internal clock through the action of neuroendocrine substances. The periodic evolution of biological processes results in internal deficits caused by the deviation of these processes from their ideal state. An example of how deficits rise in our bodies is hunger. Hunger is the deficit of feeding and increases in the absence of food. Food deprivation makes our body react by releasing ghrelin and other hormones as alerting signals that drive us to eat [34]. From this definition, and drawing on [35], Equation (6) shows how deficits rise in the biologically inspired model.

(6) $$d_{p}\left(\theta_{Z}\right)=\left|bp_{p}\left(\theta_{Z}\right)-bp_{pideal}\right|$$

Internal deficits make our bodies react and drive behavior. Behavior theories [36] suggest that the link between voluntary behavior and internal deficits is motivation. Motivation can be defined as states that urge voluntary behavior. Our model considers motivations as variables whose intensity ($m_{k}$) depends on biological deficits. Since the robot might have many biological processes with deficits, more than one motivation can have intensities different from 0. Therefore, motivations compete to become dominant and define the robot’s behavior since each motivation is related to other behavior. To avoid shallow motivations becoming dominant, we set threshold values ($t_{k}$) to limit their activation. We propose Equation (7) to define the calculation of motivational intensities.

(7) $$\begin{array}{cc} m_{k}\left(\theta_{Z}\right)=d_{p}\left(\theta_{Z}\right) & m_{k}>=t_{k}\\ m_{k}\left(\theta_{Z}\right)=0 & m_{k}<t_{k} \end{array}$$

**Figure 3:**
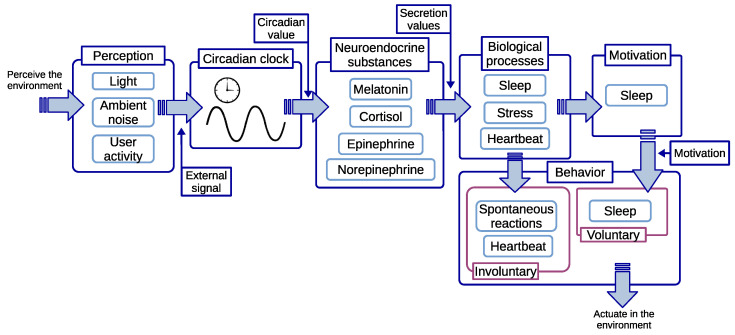
The biological model incorporates the dCiRC method to generate a circadian rhythm controlling voluntary and involuntary behavior as a reaction to ambient changes. Perception of ambient stimuli influences the velocity and timing of the clock. Then, neuroendocrine substances evolve depending on the phase and intensity of the clock affecting biological processes. The deficits of some processes affect motivation and lead to voluntary behavior, while others directly lead to involuntary behavior, like the heartbeat.

As a summary, and using Figure 3 as a detailed representation of the relationships of the model, Equations (1)–(3) produce an adaptive circadian signal (CiRC) reactive to changing ambient conditions. Then, the circadian signal influences the secretion of the neuroendocrine substances in Equation (4). The secretion of neuroendocrine substances is time-shifted on most occasions since they are not fully synchronized with the circadian clock. The values in the neuroendocrine substances generated using Equation (4) affect the value of the biological processes through Equation (5), making them evolve with time faster or slower depending on the secretion rate of the neuroendocrine substances.

The biological processes have an ideal value that makes them stay in an ideal condition (e.g., sleep’s ideal value is 0 units, meaning that the agent’s sleep pressure is null). When the value of a biological process differs from its ideal value, a deficit appears in the process (and the agent). The deficit in a biological process is calculated using Equation (6), acting as a homeostatic regulation to maintain the internal biological processes at their ideal condition through the execution of behaviors.

If a biological process has a significant deficit, a motivation rises in the agent to execute behavior that restores the internal deficit to its ideal condition (e.g., a sleep deficit will drive the sleep motivation leading the robot to sleep to restore the sleep pressure). Therefore, in the model, the deficit acts as the negative feedback in the homeostasis regulation and the execution of behaviors as the mechanism to regulate the controlled variable (the biological process).

## 4. Experimental Setup

This section describes the experimental setup we have used to show how the circadian rhythm adapts the biological processes of the agent and influences its decision-making and behavior execution. The agent we consider in this paper to integrate our model is a robot akin to our Mini social robot [37]. This robot is an embodied desktop agent with five degrees of freedom in the hip, arms, neck, and head, colored lights that emulate expressive cheeks and the heart, a speaker to talk, and expressive eyes. These devices can be modified to change the expressions they show. Besides, Mini perceives ambient stimuli using a photosensor (light), microphone (noise and user speech), touch sensors (tactile contact), and visual cues using a 3D camera.

### 4.1. Scenarios

We define the robot’s performance in different trials. We defined a circadian clock using the dCiRC model that regulates the robot’s biological functions, driving its motivation, decision-making, and behavior execution. Unlike the original definitions of the dCiRC model [10,11], the clock adapts its rhythm not only to the light profile. It considers the ambient noise and the user activity perceived by the robot. The experiments presented in this paper aimed at:

- Show how the circadian clock adapts time and angular velocity to changing environmental conditions. We demonstrate its performance in two cases: abrupt time shifts and seasonal changes in the External signal.
- Show the impact that ambient stimuli have on the circadian clock. Specifically, we show how giving more importance to the user activity can drive the robot to shift its activity to match that of the user.
- Demonstrate how the robot can dynamically adjust long-lasting voluntary behaviors like the sleep-wake cycle to external conditions that make it change its activity periods.
- Demonstrate how the circadian clock drives involuntary behaviors like heartbeat rhythm through the action of other processes like stress peak in the early morning.

The following sections define the parameters set in the biologically inspired model to simulate the dynamics of the robot’s behavior and obtain graphical results about how the robot performs in dynamic ambient conditions.

### 4.2. Circadian Definition

**Figure 4:**
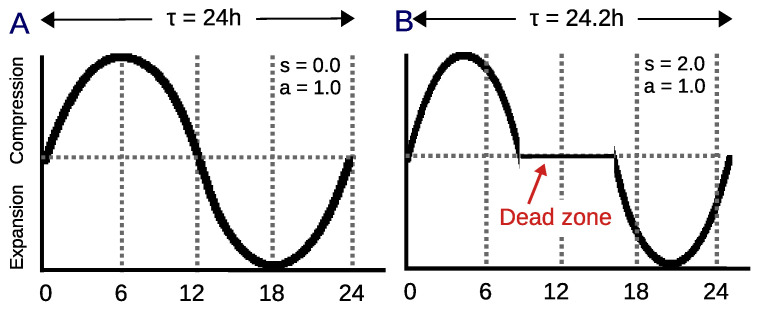
Example of two circadian rhythms generated by the model [11]. (**A**). Symmetrical circadian rhythm (s = 0.0, a = 1.0) with a period of $\tau_{I}=24$ h. (**B**). Asymmetrical circadian rhythm (s = 2.0, a = 1.0) with a dead zone in the mid-h of the day and a period of $\tau_{I}=24.2$ h.

The circadian signal employed to emulate the evolution of the biological processes in the agent follows the dynamics of the dCiRC model (see Figure 4 for two examples of different circadian clocks). In these experiments, we set the shape factor s=0 to delete the dead zone around noon and make the signal a sine function. Similarly, the asymmetry factor a=1 was selected to make the positive and negative areas of the sine function symmetrical. The External signal *T* cycle length that groups the impact of ambient stimuli on the clock and the initial internal period of the clock ($\tau_{I}$) were equally set to 24 h. The initial value of the phase angle was $\theta_{I}=0$ radians per h and the initial angular velocity ($\omega_{I}=0$) radians per h were the only values that changed during the trial. Finally, following the definition in the original dCiRC model, the stimuli strength cz=0.025, the elastic factor $k_{e}=0.05$, and the reference angular velocity $\omega_{I0}=2\pi /T$ radians per h. The original dCiRC signal ranges from −1 to 1. Our model has shifted it to be between 0 and 1 units.

### 4.3. Stimuli

The circadian clock varies depending on the ambient stimuli that the robot perceives. In this case, the model simulates the light profile, the ambient noise, and the user activity as quadratic signals ranging from 0 to 1 unit. These signals activate and deactivate at specific times of the day for 24 h. As defined in Equation (3), their values in each time step represent the value of the External signal and how strong the impact of the stimuli on the circadian clock is. The influence of each signal on the External signal depends on the weights α, β, and γ. These weights have a α,β,γ=1/3 in all the trials except when we show how user activity is more important on the External signal. In that experiment, conducted in Section 5.2, α,β=1/8 and γ=3/4 represent the higher impact of the user activity on the External signal than the light and the ambient noise as an example of how the robot activity can produce a more efficient HRI timing adapted to the user.

### 4.4. Neuroendocrine Substances

The biological model emulates four neuroendocrine substances that affect the robot’s biological processes differently. These substances are melatonin, cortisol, epinephrine, and norepinephrine. Melatonin is the primary regulator of sleep-wake cycles, so it was selected to show how the adaptive circadian rhythms autonomously control sleep and wakefulness periods. Melatonin peaks at the beginning of the sunset, causing sleep deficits and driving sleeping behavior [31]. Thus, the model includes this substance to mimic the robot’s adaptation to ambient stimuli controlling the robot’s activity periods.

Cortisol is the principal stress regulator in the human body. Unlike melatonin, cortisol peaks in the early morning, just before awakening, causing related behaviors like increased alertness and arousal [32]. Epinephrine and norepinephrine peak a few h after cortisol since they are closely associated with it, affecting important physiological functions like heartbeat [38]. The relationships between the circadian rhythms of cortisol, epinephrine, and norepinephrine are highly dependent on the circadian clock. Consequently, replicating their reactive behavior to environmental conditions can be of interest to regulate both voluntary and involuntary behavior in robots to, for example, improve their naturalness and expressiveness.

Table 2 shows the four neuroendocrine substances emulated in the biological model to obtain adaptive robot behavior from changing ambient conditions through circadian rhythms. The value of these substances has been normalized from 0.01 to 1 unit to simplify the dynamics of the model. The time shifts have been normalized to radians using the initial clock cycle length ($\tau_{I}=24$) h, being the values presented in Table 2 obtained from neuroscience studies that analyze the time at which these neuroendocrine substances present their peaks and nadirs [39].

### 4.5. Biological Processes

The biological processes emulated in this paper to demonstrate the adaptive nature of our biological model are sleep, stress, and heartbeat. They were selected to show the possibilities of the architecture to regulate processes influencing voluntary behavior through motivation (rest), involuntary physiological states that may induce escaping and alerting behaviors (stress), and reactive involuntary behavior (heartbeat). Table 3 shows how the value of the processes is calculated in each time step using Equation (5) and their ideal value that serves to calculate their deficit using Equation (6). The biological processes range from 0 to 100 units except for the heartbeat, whose value ranges from 50 to 200 beats per minute, and its average resting value (ideal value) is around 55–60 beats per min.

The relationships and values in Table 3 have been mostly empirically determined since no human study has yet measured the ranges and values of internal biological processes like sleep or stress. The only process whose values have been obtained from real human studies is the heartbeat [38] since important studies characterize this process from 50 to 200 beats per minute and resting values around 55–60 beats per min.

**Table 3:** Biological processes included in the model.

Biological Process	Basal Value (${\mathrm{bv}}_{p}$)	$\lambda_{p}$	$\delta_{p}$	Related Substances	Ideal Value (${\mathrm{bp}}_{\mathrm{pideal}}$)
Sleep	0	1	5	Melatonin	0
Stress	0	0	10	Cortisol	0
Heartbeat	60	0	10	Epinephrine Norepinephrine	60

### 4.6. Motivation and Behavior

The biological model has the *Rest* motivation to urge the robot to sleep when needed. As Equation (7) shows, the Rest motivation depends on the sleep deficit, which is the difference between the current value of the biological sleep process and its ideal value. Since motivation depends on the deficits of biological processes, motivational intensities are normalized from 0 to 100 units. To avoid the robot going to sleep if the deficit is small, a threshold value ($t_{k}$) empirically set to 50 units has been included, so the robot only sleeps if the motivation is above this value. If the sleeping behavior is active, the sleep deficit is reduced by 5 units per time step acting as a negative feedback that restores the deficit of the biological process. We calculated this value to reduce the sleep drive during the night and restore the sleeping need. Motivation is intrinsically related to voluntary behavior, so in this demonstration, the heartbeat and stress biological processes do not relate to motivation since they drive involuntary processes.

## 5. Results

The results presented in this section analyze four different mechanisms derived from the possibilities offered by the circadian clock method.

- First, we describe how the circadian clock adjusts its time and angular velocity by reacting to abrupt time shifts and simulated seasonal changes in the ambient stimuli.
- Second, we show how the circadian clock adapts to different external stimuli (light, ambient noise, and activity), showing how the clock makes the robot more aware of the user’s activity.
- Third, we show how adaptive voluntary behavior emerges following the dynamics of the biological model to control the sleep-wake cycle.
- Fourth, we present how the model deals with adaptive involuntary behavior, showing the adaptation of stress and heartbeat to ambient conditions to maintain their periodic peaks in the early morning h.

At the beginning of the trials shown in the following sections, the circadian clock is not entrained to the ambient conditions, so in most cases, the system needs 2 to 3 days to synchronize the internal processes with the ambient conditions. We support the results with animations to facilitate the visualization of the model dynamics. The animations can be watched in the following link: https://youtube.com/playlist?list=PLXNPQDsfy0lLkrUNoryHz-htgqNkfqqxx (accessed on 31 August 2023).

### 5.1. Phase Shifts and Seasonal Changes in the Circadian Clock

**Figure 5:**
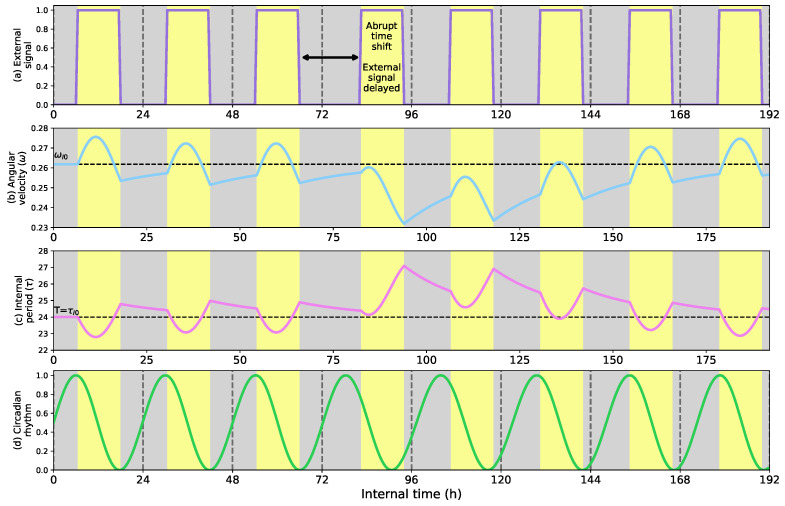
(**a**) External signal considering the light profile as external stimulus; (**b**) Angular velocity of the circadian clock (in radians per second); (**c**) Internal period of the clock (in h); (**d**) Circadian rhythm. Response of the circadian clock to an unexpected time shift delay in the light profile. At the beginning of the trial, the light profile is 12 h of light (starting at 6 a.m.) and 12 h of darkness (starting at 6 p.m.). When the time shifts occur in h 82, the angular velocity (**b**) decelerates the clock, expanding its internal period until the synchronization is completed around h 154.

Figure 5 shows how the circadian clock that controls the robots’ biological functions adapts its rhythm when an abrupt environmental change occurs. This environmental change resembles traveling between different time zones with other light profiles. Figure 5a shows the External signal that, in this case, represents the light profile (weights β,γ=0 and α=1), Figure 5b the angular velocity of the internal clock ($\omega_{I}$), Figure 5c, the cycle length of the internal clock ($\tau_{I}$) in hs, and Figure 5d the circadian rhythm of the internal clock. All graphs color light periods in yellow and dark periods in gray to facilitate the visualization of the clock’s synchronization. The trial lasts 8 24-h days.

The left side of Figure 5 shows that at the beginning of the trial, during the three first days, the light profile is equally distributed in 12 h of light (between 6 a.m. and 6 p.m.) and 12 h of darkness (from 6 p.m. to 6 a.m.). This period is characterized by synchronization since the clock adapts its rhythm to environmental conditions. The synchronization happens quickly and can be perceived in the signals of the angular velocity (Figure 5b) and clock period (Figure 5c) during the second and third days.

On the fourth day, an abrupt time shift of 4 h in the External signal (in this trial, the light profile) occurs. The time shift situates the light period from 10 a.m. to 10 p.m. and the dark period from 10 p.m. to 10 a.m. This light profile remains until the end of the trial with a fixed cycle length of the External signal of 24 h. As Figure 5b,c show, the time shift decelerates the circadian clock ($\omega_{I}$), expanding its cycle length ($\tau_{I}$). Consequently, the clock’s circadian rhythm adjusts to the light profile, situating its peak and the positive region right at dawn. As the synchronization advances, the clock’s angular velocity moves back to the initial values expanding and compressing the internal period. This example illustrates how the circadian clock adapts to unexpected time shifts in the External signal synchronizing the internal rhythm of the robot.

**Figure 6:**
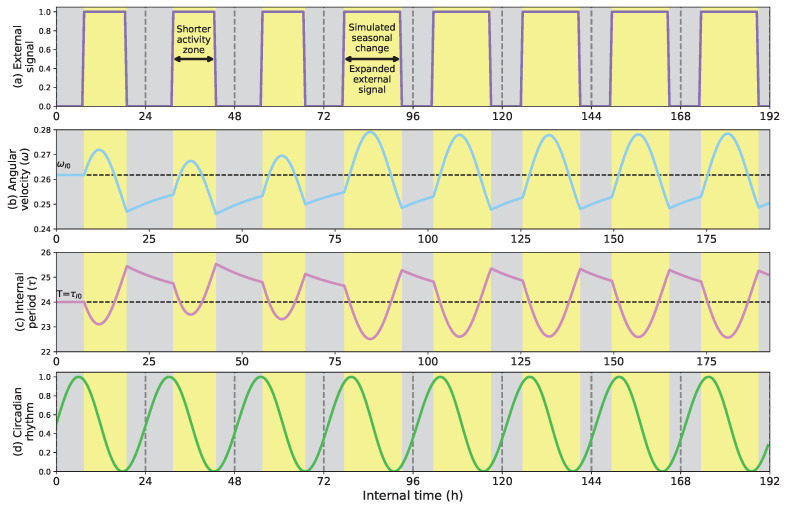
(**a**) External signal considering the light profile as external stimulus; (**b**) Angular velocity of the circadian clock (in radians per second); (**c**) Internal period of the clock (in h); (**d**) Circadian rhythm. Adaptive behavior of the circadian clock when facing a simulated seasonal change in the light profile. At the beginning of the simulation, the light h are from 6 a.m. to 6 p.m., and darkness occurs from 6 p.m. to 6 a.m. The seasonal change occurs at h 76, expanding the light h (4 more h of light) and reducing the darkness period. By reducing its velocity, the clock period expands to match the light h.

Figure 6 shows the capacity of adapting the circadian clock to seasonal changes. In this example, as in the previous case, the External signal only considers the light profile, which during the first three days is lighted from 7 a.m. to 7 p.m. and dark from 7 p.m. to 7 a.m. Then, from the fourth day on, we simulate a seasonal change by expanding the light h from 5 a.m. to 9 p.m. (16 h long). This change in the light profile emulates the extended light h that typically occur in summer, increasing the circadian clock’s velocity significantly (see Figure 6b) during the light h and reducing it during the dark periods. As a consequence, the positive region of the circadian signal expands, and the negative area compresses (see Figure 6d). The imbalance of the positive and negative regions of the circadian clock might, for example, explain changes in mood and emotion in darker areas (arctic pole in winter) compared to lighter ones (Spain in summer) [40].

The results presented in this section show how the circadian clock dynamically adapts to abrupt and seasonal changes in the light profile (the only stimulus considered in the External signal). However, this adaptation can be extrapolated to other situations in which the External signal considers other external stimuli, such as ambient noise or user activity. As the following section shows, these stimuli have been identified as important markers in the human circadian clock, so adapting to them is essential to maintain a balance in biological processes such as social interaction.

### 5.2. Influence of Ambient Stimuli on the Clock

**Figure 7:**
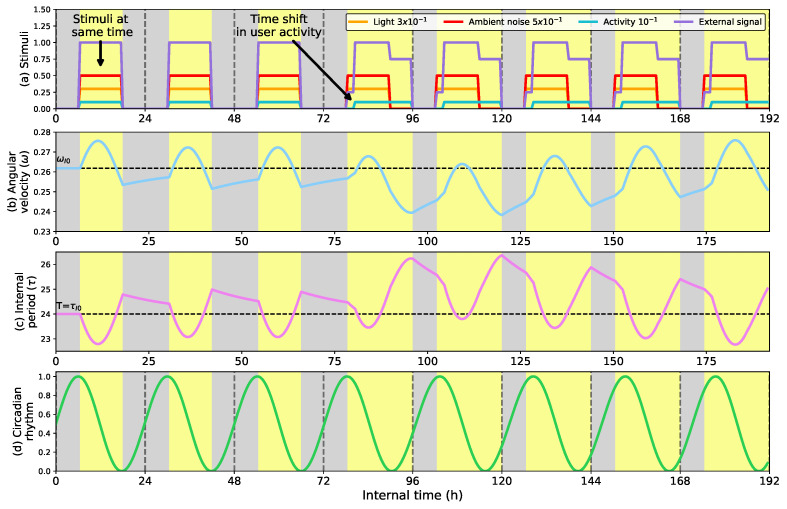
(**a**) External signal calculated using Equation (3) with α=1/8, β=1/8, and γ=3/4 and the intensity of each stimulus; (**b**) Angular velocity of the circadian clock (in radians per second); (**c**) Internal period of the clock (in h); (**d**) Circadian rhythm. Adaptive response of the circadian clock to different ambient conditions by considering the user activity periods as the primary stimulus that the clock considers for adaptation. Since the user activity is the primary marker of the circadian clock, when the user shifts and expands the activity (in h 80) from 8 a.m. to midnight, the circadian signal adapts to this change.

Unlike the previous two examples where the External signal is only affected by the light conditions, Figure 7 shows the adaptation of the circadian clock to dynamic conditions when the External signal considers the light profile, ambient noise, and user activity. In this example, the External signal considers the most powerful stimulus of the user activity (setting the weights of the External signal empirically to α=1/8, β=1/8, and γ=3/4), simulating that the robot adapts its activity to obtain a more efficient HRI. The External signal distribution is shown in Figure 7a.

In the first three days, the ambient stimuli (light, ambient noise, and user activity) present the same periodicity. They are active from 6 a.m. to 6 p.m. and inactive the rest of the time. On the fourth day, the user activity moved two h forward and extended being active from 8 a.m to midnight. This movement changes the value of the External signal, affecting the activity periods of the robot. Given this situation, the circadian clock reduces its velocity by expanding its cycle length and synchronizing its time peak with the user activity (the most important stimulus). Figure 7d shows the adaptation of the circadian clock to the user activity, also considering the light profile and the ambient noise but to a lesser extent. This result shows how the robot activity adjusts to the most important stimulus. For example, in social robots, the robot activity can be adapted to that of their users, while in other kinds of robots, other stimuli can act as the primary stimulus.

This section shows a potential application of the model to synchronize the robot and the user activity for a more efficient HRI. The External signal considers the user activity as the most important marker of the robot’s circadian clock, matching its activity to the user. The model we propose in this contribution can be focused on other stimuli and applications, generating a robot behavior adapted to different situations. The following sections show the adaptive behavior of the model to yield voluntary and involuntary behavior in robots oriented to interacting with people, regulating their activity, and involuntary expressiveness during the day.

### 5.3. Controlling Cyclic Behavior: Sleep-Wake Case

The circadian rhythm generated by the dCiRC model aims to control the artificially created biological functions of the robot. The sleep-wake cycle regulates its activity periods, preventing the robot from always being active. This section shows how the robot can autonomously manage the user activity to match the ambient conditions in two different situations: abrupt changes in the active period of the External signal and expansion of the activity periods that may occur in different seasons.

**Figure 8:**
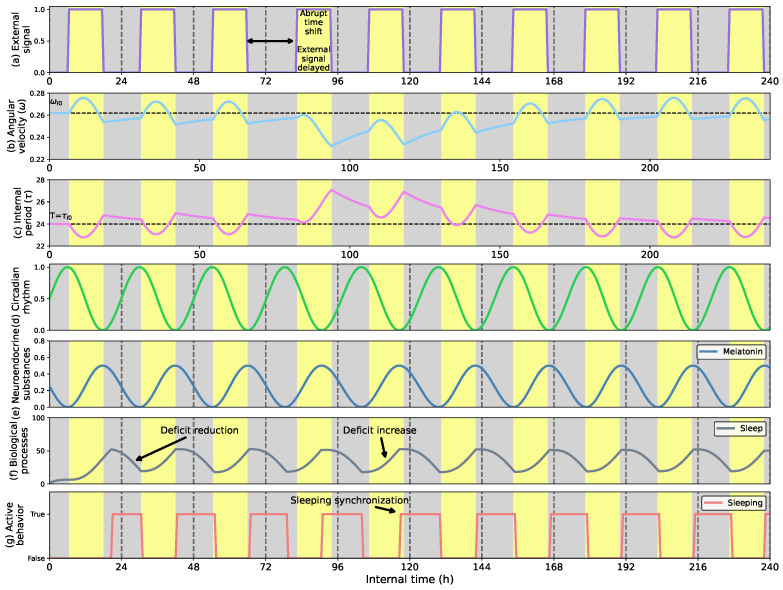
(**a**) External signal considering the light profile as external stimulus; (**b**) Angular velocity of the circadian clock (in radians per second); (**c**) Internal period of the clock (in h); (**d**) Circadian rhythm; (**e**) Melatonin rhythm; (**f**) Sleep process; (**g**) Sleeping behavior. The sleep-wake regulation during ten consecutive days is an action of the adaptive circadian rhythm and the melatonin effects on the sleep drive. When an abrupt time shift occurs, the robot can adapt its wake-sleep cycle thanks to the adaptive response of the circadian clock and the synchronization of the melatonin secretion.

Figure 8 shows the adaptive behavior of the robot to regulate its sleep-wake cycle to match the external conditions. As in the previous cases, Figure 8a shows the External signal distribution, Figure 8b the clock’s angular velocity ($\omega_{I}$), Figure 8c the internal period ($\tau_{I}$), Figure 8d the circadian clock evolution, Figure 8e the melatonin rhythm, Figure 8f the biological sleep process, and Figure 8g when the sleeping behavior activates showing that the robot is sleeping.

The distribution of the External signal starts with 12 h of activity between 6 a.m. and 6 p.m. and 12 h of inactivity between 6 p.m. and 6 a.m. Then, on the fourth day, the activity period moves forward 4 h, situating the activity periods from 10 a.m. to 10 p.m. and the inactivity period from 10 p.m. to 10 a.m. The time shift decelerates the clock synchronizing the melatonin rhythm to the activity period (see Figure 8e). Since melatonin affects the evolution of the biological sleep process, its accumulation drives increased sleep pressure, as Figure 8f shows. Finally, the sleep deficit affects the robot’s motivation to sleep. When the motivation is above the threshold value of 50 units, it leads the robot to rest and reduces the deficit. As Figure 8f,g show, the biological mechanisms synchronizes the robot’s activity to the external activity, autonomously regulating the periods in which the robot operates.

**Figure 9:**
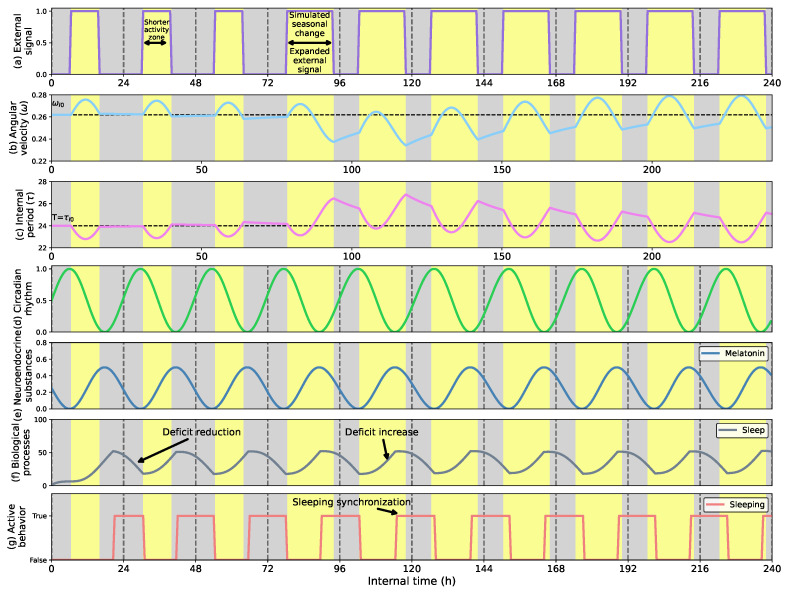
(**a**) External signal considering the light profile as external stimulus; (**b**) Angular velocity of the circadian clock (in radians per second); (**c**) Internal period of the clock (in h); (**d**) Circadian rhythm; (**e**) Melatonin rhythm; (**f**) Sleep process; (**g**) Sleeping behavior. The sleep-wake expansion and compression to seasonal changes adapt the activity h of the robot. A seasonal change in the light profile leads to a broader circadian signal that expands the activity h of the robot and reduces the time it spends sleeping.

Figure 9 shows how the adaptive circadian clock can compress and expand the robot’s sleep cycle to match the external activity h. As Figure 9c shows, during the first three days of the trial, the outer activity is from 6 a.m. to 4 p.m. The external activity period compression reduces the clock’s cycle length (see Figure 9b), leading the robot to synchronize and increase its resting h. On the fourth day, the external activity suffers an abrupt expansion of 8 h, situating the external activity from 6 a.m. to 10 p.m. This change leads the robot to shift its sleep-wake cycle and reduce the resting h, making it more active. However, as Figure 9f shows, the definition of the biological model leads the robot not perfectly to fit the sleeping h to the external profile.

### 5.4. Arousal Effects and the Circadian Clock

**Figure 10:**
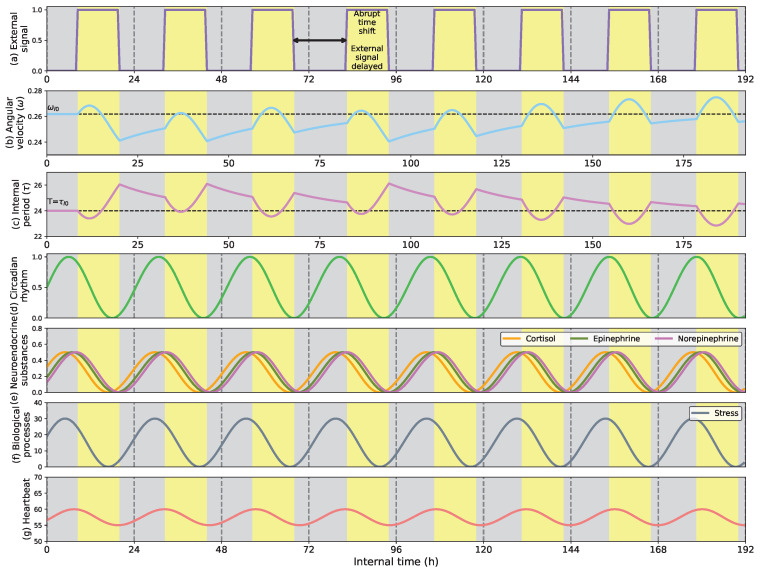
(**a**) External signal considering the light profile as external stimulus; (**b**) Angular velocity of the circadian clock (in radians per second); (**c**) Internal period of the clock (in h); (**d**) Circadian rhythm; (**e**) Neuroendocrine rhythms; (**f**) Stress process; (**g**) Heartbeat evolution. The stress and heartbeat adaptation to abrupt time shifts maintain their peak values at the first h of the activity synchronizing the internal biological processes of the robot to ambient conditions.

**Figure 11:**
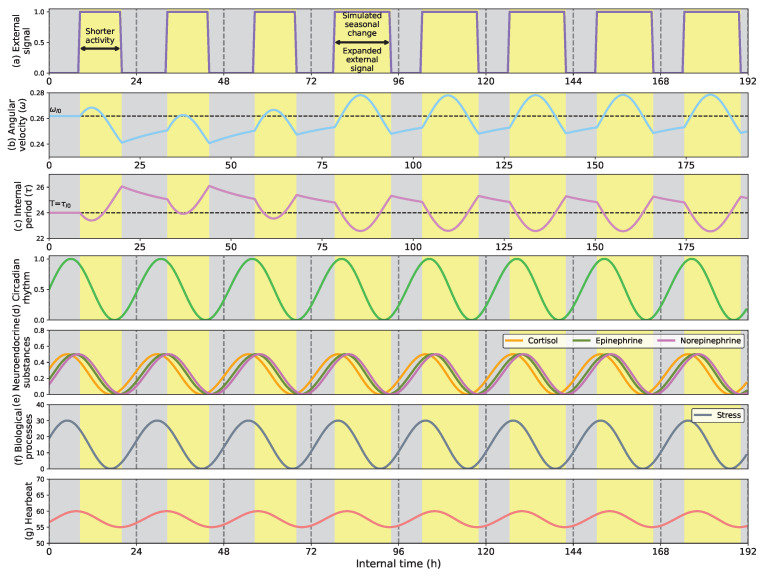
(**a**) External signal considering the light profile as external stimulus; (**b**) Angular velocity of the circadian clock (in radians per second); (**c**) Internal period of the clock (in h); (**d**) Circadian rhythm; (**e**) Neuroendocrine rhythms; (**f**) Stress process; (**g**) Heartbeat evolution. The stress and heartbeat adaptation to expanded activity h occurs at specific seasons of the year, like summer. In the robot, this change causes an expansion of the stress levels and heartbeat values, increasing alertness and arousal states.

The biological model based on biological rhythms regulates long-lasting behavior like the sleep-wake cycle and other essential physiological functions that might be interesting to emulate in robots. To illustrate this application, Figure 10 and Figure 11 show the adaptive regulation of the stress and heartbeat processes to the ambient condition through the action of cortisol, epinephrine, and norepinephrine. These functions peak in the early morning affecting many involuntary functions to manage arousal and activation, so emulating them in robots could be of particular interest to improve the involuntary reactions of robots socializing with people.

Figure 10 shows the adaptive regulation of the stress and heartbeat processes to abrupt time shifts. At the beginning of the trial, the clock is not entrained, so it needs some days to fit into the new ambient profile. In this case, the External signal represents the light-dark h, simulating during the first three days a light profile of 12 h light (from 8 a.m. to 8 p.m.) and 12 h darkness (from 8 p.m. to 8 a.m.). Then, the light moves forward two h, situating the light period from 10 a.m. to 10 p.m.

Figure 10f,g show that the stress and heartbeat peaks synchronize with the first h of the light period. Besides, it is possible to perceive that the heartbeat peak is about two h later than the stress peak. This fact is due to the dependency of stress on cortisol (that peaks right after dawn) and the dependence of heartbeat on epinephrine and norepinephrine (that peak a couple of h after cortisol since they are synthesized from it). This synchronization can derive from reactive responses during the morning, showing similar human rhythms that might be interesting in studying involuntary behavior.

If the light profile is extended, like in Figure 11, the results are similar to the ones obtained in the previous trials. The cortisol rhythm adjusts to present its peak in the early morning. However, due to the circadian signal’s expansion, the stress process’s positive region is also expanded (see Figure 11f). Similarly occurs with the heartbeat rhythm (see Figure 11g), which peaks a couple of h after dawning and is subtly expanded due to the longer duration of the positive region of the circadian clock as a response to the extended period of the light profile. Although the expansion of the stress and heartbeat processes might not be easily perceived in Figure 11f,g, the increased angular velocity of the clock (shown in Figure 11b) and the period of the internal clock shown in Figure 11c permit to visualize that during the light h, the velocity increases extending the cycle length of the clock.

## 6. Discussion

The following sections delve into the primary outcomes of the adaptive processes exhibited by the circadian clock when facing different situations. Besides, we discuss the main limitations of the biological model, providing possible solutions to address in future work.

### 6.1. Adapting the Master Clock

Robots operating in dynamic environments and interacting with different people require adaptive mechanisms to orient their behavior to the situation and user features successfully. Adapting to the ambient condition is only possible if robots can perceive the changes using their embodied sensors. Once stimuli are correctly perceived, adaptive mechanisms are necessary to produce broad behavior matching each situation.

Robots emulating human behavior to exhibit natural behavior and improve the user’s perception and trust of these systems imply that the adaptive mechanisms should also be biologically inspired. In this context, including a circadian master clock mimicking how humans regulate their behavior throughout the day might lead robots not only to provide a biological basis but enable the replication of human-like processes to improve the robot’s capabilities.

The results above demonstrate the powerful capacities of the dCiRC method to generate adaptive circadian rhythms. Combining this clock with the biological functions emulated in our model permits robots to generate behavior like humans, possibly making their users perceive the robots as more human-like and capable, leading to increased trust. However, this method needs to be evaluated in human-robot interaction studies.

### 6.2. Regulating Long-Lasting Behavior

The model presented in this paper allows robot control in long-lasting operation. Nowadays, most robotic platforms only operate in controlled settings performing predefined behavior to solve specific and punctual tasks. Suppose robots are to be deployed in broader and more complex scenarios where unpredictable situations occur. In that case, they should incorporate mechanisms to recognize the current state of the environment and autonomously generate appropriate responses without restarting the system.

The results show the model’s adaptation and control of the robot’s activity to that of its users through the sleep-wake cycle. The trials last from 8 to 10 days, verifying that our controller yields adaptive behavior during long periods. However, the model definition requires predefined parameters related to neuroendocrine substances, biological processes, the clock’s adaptive behavior, or the effects of the behavior. As Figure 9 shows, sometimes, these parameters influence the robot’s behavior. For example, although the robot adapts its sleeping behavior to the activity periods, its effect (set to reduce the sleeping deficit in 5 units per time step) can lead the robot to sleep more than necessary since this value is set empirically. The robot needs more resting h than the external activity provides, so it takes the end of the activity period and the beginning of the day to sleep and reduce its internal deficit appropriately. Therefore, we should explore new methods to avoid predefined parameters in the model.

This paper provides examples showing the model possibilities and how to endow robots with adaptive biologically inspired behavior. We show the evolution of some functions to show the model’s operation. However, designers can integrate other biological functions to extend the robot’s functions, for example, execute certain activities like entertaining the user, alerting about insecure situations, or notifying important events such as doctor appointments. Consequently, generating biologically inspired behavior requires including more processes in the loop, endowing the robot with a broader number of voluntary and involuntary behaviors that extend the number of tasks the robot can solve. Among the possible voluntary behavior that designers can integrate into the model, a few examples could be social and affective interaction, entertainment, or motivational navigation. Regarding involuntary behavior, our model can automatically control other autonomic processes like blinking, breathing, or locomotor activity, especially in embodied interactive robots [27].

### 6.3. Limitations

The biological model presented in this contribution contains some limitations worth mentioning. Next, we enumerate them and attempt to provide a solution we would like to address in further tests.

- **Model parameters:** The primary drawback of the biological model presented in this paper is the high number of parameters that play a role in the circadian clock and the rest of biological functions like neuroendocrine substances and biological processes. These parameters affect the model performance and, therefore, the behavior exhibited by the robot, so obtaining them from real studies can be an alternative to produce more realistic biologically inspired systems.
- **Scalability:** Modeling biologically inspired phenomena in artificial agents like robots requires precise synchronization tuning. The emulation of biological processes affects the system’s scalability, increasing its complexity and dedication as the number of processes grows. This problem is important because as the number of robot behaviors increases, the users’ perception also improves. To overcome this limitation, it is essential to develop general methods that simplify the definition of the biological functions proposed in the robot. This paper proposes new advances like using the dCiRC model and methods to characterize motivation, neuroendocrine substances, and other biological processes from this rhythm.
- **Robot hardware requirements:** We would like to integrate and evaluate the model using our Mini social robot [37] to endow it with more natural and adaptive behavior. Mini possesses sensors and actuators to perceive stimuli and execute behavior. However, their performance can be affected if the model is applied to other robots or systems lacking the necessary hardware devices. We believe that the sensors for perceiving ambient light intensity or noise are widely available nowadays, so robots can easily integrate them into other robots to use the model.
- **User evaluation:** The generation of biologically inspired and adaptive behavior needs an evaluation from the users. In the future, we plan to extend the number of processes integrated into the model to obtain the users’ impressions of the robot’s performance.

## 7. New Insights and Potential Applications

The results presented in this paper show how the biological model proposed in this contribution regulates voluntary long-lasting behavior (sleep-wake cycle), biological processes with significant effects at specific h of the day (stress) that might drive to execute behavior, and involuntary processes like the heartbeat that are necessary to convey the robot’s state to their users. However, the model’s possibilities are broader than adapting to time shifts and seasonal changes. As we demonstrated, changing the way dCiRC considers the ambient condition (only considers light profile) by incorporating other signals like the ambient noise or the user activity as an indicator of the periods that people use the robot allows adapting the robot behavior to the user activity.

This work’s most important future work is integrating the model into a real robot and evaluating if its biological behavior changes the users’ perceptions of some robot attributes, such as naturalness, and impacts HRI. To attain this goal, it is necessary to increase the number of human studies that support our model to provide a more accurate approximation of the emulated biologically inspired functions of the model. A good starting point could be extending the number of biological processes using predefined model parameters of the robot emulating, for example, a child and including goal-directed tasks in HRI.

Furthermore, we expect to apply the model in other interesting domains like affective computing. Studies in neuroscience suggest that ambient conditions affect our mood and emotional states [40]. Besides, the substances regulating human affection (dopamine, serotonin, or oxytocin) experience circadian rhythms, so their secretion varies throughout the day, affecting our affective state. Considering these studies, we would like to study the variability of affective states along the day and possible disorders in mood and emotion caused by seasonal changes or abrupt shifts in the external environment.

Circadian rhythms are not the only periodic control that exists in humans. Ultradian rhythms are periodic variations whose period is below a day. For example, biological processes affected by ultradian rhythms are hunger, thirst, cortisol peaks in the early morning after awakening, or changes in blinking frequency. In the literature, we have und to find a biological model that aims to shape the dynamic nature of ultradian rhythms. For this reason, in future work, we find it interesting to explore and include the impact of ultradian rhythms on robot behavior in our biological model.

The last point we want to investigate is how generating involuntary behavior to respond to unexpected environmental situations can improve human-robot interactions. Most research in autonomous robots has been directed at creating robots with voluntary behavior, not exploring the important role of involuntary behavior in social interaction and communication. We want to take advantage of this niche and study whether involuntary behavior can improve human-robot interactions by facilitating users to perceive and understand the actions the robot is executing.

## 8. Conclusions

This paper describes how applying the circadian clock proposed in the dCiRC model [10] can be used to extend the adaptive capabilities of robots operating in dynamic environments by perceiving the changes in the ambient stimuli and adapting to them. The model mimics how human rhythms emerge and adapt to ambient conditions, providing a realistic way of exhibiting biologically inspired functions that are especially important for human-robot social interactions and autonomous behavior.

The possibilities provided by the model raise the number of biologically inspired behaviors that the robot can show, improving its interaction capabilities and adaptation to the environment. As discussed in the previous sections, the model can be applied to more complex scenarios, enhancing the repertoire of voluntary activities of the robot and other aspects of behavior like involuntary actions. 

## Figures and Tables

**Table 1:** Abbreviations used by the model, the equation of the model where they are used, their meaning, ranges, and units.

Abbreviation	Model Use	Meaning	Range and Unit
$\theta_{Z}$	Circadian clock	Phase of the external clock	[0,2π] radians
$\theta_{I}$	Circadian clock	Phase of the internal clock	[0,2π] radians
CiRC	Circadian clock	Circadian value in time $\theta_{Z}$	[0, 100] units
*s*	Circadian clock	Shape factor	[0,2] units
*a*	Circadian clock	Asymmetry factor	[0,2] units
$\tau_{I}$	Circadian clock	Period of the internal clock	(0,24] h
*T*	Circadian clock	Period of the External signal	(0,24] h
$\omega_{I}$	Circadian clock	Angular velocity of the internal clock	(0,2π/24] radians per h
$\omega_{I0}$	Circadian clock	Angular velocity in constant darkness	(0,2π/24] radians per h
cz	Circadian clock	Impact of the stimuli on CiRC value	(0,1] units
ke	Circadian clock	Elastic factor to correct the angular velocity	(0,1] units
External signal	Circadian clock	Intensity of stimuli	[0,100] units
α	External signal	Impact of light on clock	[0,1] units
β	External signal	Impact of noise on clock	[0,1] units
γ	External signal	Impact of the robot activity on clock	[0,1] units
light	External signal	Ambient light intensity	[0,100] units
noise	External signal	Ambient noise intensity	[0,100] units
activity	External signal	User activity value	[0,100] units
$ns_{n}$	Neuroendocrine subs.	Value of neuroendocrine substance *n*	[0,100] units
$\kappa_{n}$	Neuroendocrine subs.	Impact of CiRC value on substance *n*	[0,1] units
$\theta_{n}$	Neuroendocrine subs.	Phase shift of substance *n*	[0,2π] radians
$bp_{p}$	Biological processes	Value of biological process *p*	[0,100] units
$bv_{p}$	Biological processes	Basal value of process *p*	[0,100] units
$\lambda_{p}$	Biological processes	Accumulative effect of process *p*	0 or 1 unit
$\delta_{p}$	Biological processes	Impact of substance *n* on process *p*	[0,1] units
$d_{p}$	Deficit	Deficit of a biological process *p*	[0,100] units
$bp_{ideal}$	Deficit	Ideal value of a biological process	[0,100] units
$t_{k}$	Motivation	Threshold value for motivation *k*	[0,100] units
$m_{k}$	Motivation	Intensity of motivation *k*	[0,100] units

**Table 2:** Neuroendocrine substances included in the model.

Neuroendocrine Substance	$\kappa_{n}$	Phase Shift ($\theta_{n}$)	Effects
Melatonin	0.25	12 h × 2 π / $\tau_{I}$	Induces sleep
Cortisol	0.25	23 h × 2 π / $\tau_{I}$	Stress regulation
Epinephrine	0.25	1 h× 2 π / $\tau_{I}$	Increased heartbeat
Norepinephrine	0.25	2 h× 2 π / $\tau_{I}$	Increased heartbeat

## Data Availability

Data sharing not applicable.
